# Epidemiology and Clinical Characteristics of Influenza C Virus

**DOI:** 10.3390/v12010089

**Published:** 2020-01-13

**Authors:** Bethany K. Sederdahl, John V. Williams

**Affiliations:** 1 Department of Pediatrics, University of Pittsburgh School of Medicine, Pittsburgh, PA 15213, USA; Sederdahl.Bethany@medstudent.pitt.edu; 2 Institute for Infection, Inflammation, and Immunity in Children (i4Kids), University of Pittsburgh, Pittsburgh, PA 15224, USA

**Keywords:** orthomyxoviruses, influenza C, epidemiology

## Abstract

Influenza C virus (ICV) is a common yet under-recognized cause of acute respiratory illness. ICV seropositivity has been found to be as high as 90% by 7–10 years of age, suggesting that most people are exposed to ICV at least once during childhood. Due to difficulty detecting ICV by cell culture, epidemiologic studies of ICV likely have underestimated the burden of ICV infection and disease. Recent development of highly sensitive RT-PCR has facilitated epidemiologic studies that provide further insights into the prevalence, seasonality, and course of ICV infection. In this review, we summarize the epidemiology and clinical characteristics of ICV.

## 1. Introduction

Influenza C virus (ICV) is lesser known type of influenza virus that commonly causes cold-like symptoms and sometimes causes lower respiratory infection, especially in children <2 years of age [1]. ICV is mainly a human pathogen; however, the virus has been detected in pigs, dogs, and cattle, and rare swine–human transmission has been reported [2,3,4,5,6]. ICV seropositivity has been found to be as high as 90% by 7–10 years of age, suggesting that most people are exposed to influenza C virus at least once during childhood [7,8]. Although ICV was discovered in 1947, disease burden has been poorly described until recently due to difficulty isolating the virus in cell culture [9,10,11,12,13]. Human challenge studies confirmed that ICV caused upper respiratory disease and immune responses [14]. The recent development of RT-PCR for ICV detection has resulted in expanded understanding of ICV clinical characteristics, seasonality, and molecular epidemiology. A related novel influenza D virus (IDV), was discovered in swine in Oklahoma in 2011 and has been detected in other mammals [15,16,17,18,19]; however, while cattle workers exhibit seropositivity, no definitive evidence of productive human infection with IDV has been reported [20,21]. In this review, we discuss the epidemiology and clinical characteristics of ICV.

### Virus Structure

ICV is an enveloped, negative-sense RNA virus that belongs to the *Orthomyxoviridae* family. It has a 7-segmented genome that encodes 9 viral proteins [22,23,24], distinguishing it from influenza A and B viruses that have 8-segment genomes encoding 10 major viral proteins [25]. Some IAV strains express other proteins PB1-F2 or PA-X from alternate reading frames [26,27]. The presence of a single surface glycoprotein that combines the function of two surface proteins found on influenza A and B viruses is another key feature that distinguishes ICV from influenza A and B viruses [28,29,30]. Influenza A and B surface proteins include hemagglutinin (HA) and neuraminidase (NA), which mediate attachment, entry, and escape [25,31]. In contrast to influenza A and B, ICV hemagglutinin-esterase-fusion (HEF) glycoprotein, encoded on segment 4, efficiently fulfills the roles of both HA and NA by facilitating host receptor binding, cleaving sialic acid, and membrane fusion [32,33,34,35]. However, ICV HEF binds to N-acetyl-9-O-acetylneuraminic acid rather than to N-acetyl-neuraminic acid for influenza A and B viruses [36]. HEF is the major target for host neutralizing antibodies, which appear to bind to epitopes near the receptor-binding site and the esterase site [37,38,39,40,41,42]. Human CD8+ T cells recognize epitopes of ICV internal proteins, some of which are conserved in IAV and IBV [43].

M1, encoded from segment 6, is the major structural protein of ICV that lies under the lipid bilayer [44,45]. The internal structure of ICV is dominated by ribonucleoproteins (RNPs) that are composed of ribonucleic acid and four structural proteins. Genome segment 5 codes for nucleoprotein (NP) and segments 1–3 code for the polymerase (P) subunits basic (PB)2, PB1, and P3, respectively [44,45,46]. Segment 6 also encodes CM2 protein, a minor envelope glycoprotein ion channel [47]. Segment 7 encodes Non-structural protein 1 (NS1), which inhibits host immune responses and Nuclear Export Protein (NEP), which mediates nuclear export of viral RNP [48,49,50,51,52,53]. Like other influenza viruses, ICV viruses have a segmented genome capable of reassortment; reassortment has been documented in vitro as well as in vivo among circulating strains [54,55,56,57,58].

## 2. Epidemiology and Clinical Characteristics

### 2.1. Methods of Detection

Seropositivity studies have provided key insights into the epidemiology of ICV but have several limitations including limited ability to determine time of infection. This makes it difficult to identify active infection, describe symptoms, isolate virus for molecular epidemiology, or determine seasonality. Recent epidemiologic studies have taken advantage of improved cell culture techniques and RT-PCR as a means of detecting ICV and have provided further insight into the characteristics of active ICV infection. Until recently, cell culture has been used as the primary method of detecting ICV cases and outbreaks, including many studies in Japan [1,58,59,60,61]. However, the weak cytopathic effect of ICV makes it difficult to detect, resulting in underestimation of burden [10,11,12,13,62]. Seroepidemiology studies of ICV infection measuring hemagglutinin inhibition (HAI) antibody titers have been key in demonstrating the widespread nature of ICV circulation and infection. Within the last decade, highly sensitive nucleic acid detection (RT-PCR) methods have been developed for the detection of ICV [63,64]. In a study comparing RT-PCR to cell culture detection of ICV, RT-PCR detection rate was nearly twice that of cell culture and samples with lower viral load were more likely to be detected with sensitive nucleic acid methods [64]. Several RT-PCR assays have been reported, with significantly increased sensitivity compared to culture [63,64,65,66,67,68,69,70]. These molecular assays have facilitated epidemiologic investigations of ICV.

### 2.2. Seroepidemiology

In the decade following initial recognition of ICV, studies reporting ICV outbreaks and seroprevalence suggested that ICV infection was widespread among children in the US and England [71,72,73]. Seropositivity studies have demonstrated that ICV has an extensive global distribution and is acquired during childhood, although the age of primary infection may vary [73]. A Japanese study including 434 individuals showed seropositivity of 100% among infants <6 months old, presumably maternally derived, dropping to a nadir by 6 months. Increases in ICV seroprevalence began to rise notably by one year of age and by age 7–10 years, 80–90% of children were seropositive [8]. A California group reported an ICV outbreak that was first detected among healthcare workers and tested 334 serum samples from participants <1 to 25 years of age. Seropositivity increased with age, with 64% seropositivity in children 0–5, 96% in children 6–10, and 98% in adults 16–25 years. Stability of HAI titer across age groups suggested periodic reinfection that maintained antibody titers [74].

Similar findings were reported in another US study that included sera from 237 subjects in 4 age groups: 1 to 2 years (36%), 2 to 5 years (47.2%), 20 to 30 years (96%), and 65 to 85 years (66.7). The highest level of seropositivity was found among young adults (20–30 years), while low ICV HAI titers and decreased seropositivity in those 65–85 years of age may suggest waning ICV immunity in the elderly [7]. These findings were supported by a French study of 301 subjects. HAI antibodies were detected in 61% of samples overall with the highest rate of seropositivity found among those 16–30 years of age (76%), while young children (<15 years) and older adults (51–88 years) had lower rates of seropositivity (46% and 44% respectively) [75]. A Spanish study including 191 subjects 1 to 80 years of age living showed seroprevalence of 68% [76].

Studies conducted in India, Jamaica, Japan, the Philippines, and other countries corroborate the widespread nature of ICV and general age distribution already described [77,78,79,80,81,82,83]. Collectively, these data indicate that ICV infection is widespread globally with most infections occurring in young children. ICV is uncommon in hospitalized adults but has caused outbreaks in military recruits [84,85,86,87,88]. ICV has been reported among travelers on the Hajj pilgrimage [89].

### 2.3. ICV in Children

A number of studies have focused on pediatric populations. As noted above, seroepidemiology shows that the majority of primary ICV infection occurs during early childhood. An early study from Japan noted most patients were around one year old [12], while reports from the UK detected ICV almost exclusively in children [11,90]. A Japanese longitudinal study of 190 ICV isolates collected over seven years found that nearly all were <6 years old, with the highest rates of infection in children 1–2 years old [60]. Rates of detection of ICV in outpatient or hospitalized children with acute respiratory illness have ranged from 0.7–10% in studies from Australia, Canada, Cuba, India, Italy, Japan, Nigeria, Peru, Scotland, and Spain [1,12,20,61,63,68,70,91,92,93,94,95,96,97,98,99,100,101,102]. Most of these studies found higher rates of ICV infection in younger children. Several studies have reported ICV as a cause of radiographic pneumonia in children, in some series as frequently as IBV [92,95,102]. These widely varied rates are likely due to varying circulation of ICV in different years; several studies have reported large outbreaks in a single year [61,94,98,99]. Outbreaks have been reported in long-term pediatric residential facilities and schools [68,91].

### 2.4. Seasonality

Seasonality of ICV is poorly understood, although outbreaks and cases of familial transmission have been described [74,101]. Matsuzaki et al. found in a multi-year Japanese study that the peak of ICV was in May during biennial epidemics in even-numbered years [60]. Gouarin et al. reported a peak of disease in France in winter-spring of 2005 while little ICV was detected in the two following seasons [101]. Fritsch et al. noted a similar seasonal pattern in Germany, with a peak of ICV detection in fall–winter–spring of 2012–2013, with minimal detection in the seasons preceding and following [99]. Thielen et al. describe a winter-spring outbreak of 51 cases in the US during 2013–2015 while in the seasons before and after only 2 and 8 cases were reported, respectively [103]. A single-year study performed by Pabbaraju et al. also identified a winter-spring seasonality in ICV detection [70]. In most studies, winter–spring seasonality remained consistent, though Anton et al. report year-round detection of ICV in Spain with highest numbers observed in the summer [104].

Population immunity may contribute to the variability of ICV. Substantial antigenic and genetic diversity exists among ICV isolates; there are six genetic lineages representing six antigenic groups of HEF, with two major genetic lineages of the internal genes [56,57,58,59,60,87,105,106,107,108,109,110,111]. Elegant longitudinal studies in Japan that compared the antigenic and genetic character of circulating isolates with concurrent serology showed periodic epidemics of ICV every few years. While multiple strains co-circulated, there was a dominant antigenic group that was replaced every few years, driven by herd immunity [56,60]. However, there was very little antigenic drift over time [56].

### 2.5. Clinical Characteristics

ICV is usually associated with mild respiratory disease. The most common symptoms associated with ICV infection are fever, rhinorrhea, and cough; however, the virus has been associated with pneumonia, bronchiolitis, and bronchitis [1,12,63,77,92,94,95,101,102,103,104]. Symptoms of gastroenteritis in patients infected with ICV are frequently reported (Table 1).

ICV infection is more commonly associated with hospitalization and lower respiratory disease in young children (Table 1). ICV-associated hospitalization occurs most often among children <3 years of age and cases of Intensive Care Unit admission among infants with prematurity and congenital heart disease have been described, as well as otherwise healthy young children [1,100,103]. Among children hospitalized for ICV infection, co-morbidity is reported in 58–80% of cases [1,103]. Prematurity is the most common comorbidity present in ICV-associated hospitalization; however, asthma, IgG deficiency, acute lymphoblastic leukemia, cystic fibrosis, and congenital heart disease have also been described [1,103].

Co-infection with other microbes is a common finding among patients with ICV infection, especially among those <2 years (Table 2). Rates of co-infection with at least 1 additional pathogen are reported in 8–50% [1,103]. Co-infection may be associated with increased severity of disease. Thielen et al. reported 3 of 5 ICV-positive patients admitted to the ICU with co-infections [103].

Respiratory viruses can interact with each other or bacteria affecting predisposition to severe respiratory disease, particularly in patients with underlying immunodeficiency or chronic respiratory disease such as chronic obstructive pulmonary disease or cystic fibrosis [112,113]. The presence of influenza or other community-acquired viruses can compromise physical and immunologic barriers and increase the likelihood of secondary bacterial infection [112]. The possible role of ICV in bacterial–viral or viral–viral respiratory co-infection is of interest but is not well understood. In a study of 706 infants <2 years old hospitalized with respiratory illness, 6 patients had influenza C virus infection, and 3 were co-infected with RSV or adenovirus [92]. While the low number of patients hospitalized with ICV and co-infection with another respiratory pathogen may minimize the role of ICV in respiratory infection leading to hospitalization, few studies have been performed and uncertainty remains.

## 3. Conclusions

ICV is an important respiratory pathogen of childhood, though there are wide variations in prevalence from year to year and in different regions. While the most common manifestation of ICV infection is upper respiratory infection, severe lower respiratory infection does occur. Co-infection with other viral and bacterial pathogens is frequent, making the causal role of ICV in these cases uncertainty. Larger scale studies describing year-to-year prevalence, clinical characteristics, and strain type are needed. ICV exhibits minimal antigenic drift over time, suggesting that a monovalent vaccine could be effective against childhood infection.

## Figures and Tables

**Table 1:** Summary of clinical characteristics of patients infected with influenza C virus.

Manuscript	Location	Age Range	Median Age (yrs)	N	Hospitalized	Fever (%)	Rhinorrhea/Rhinitis	Cough	Wheeze	Headache	Diarrhea	Vomiting	Lower Resp
Moriuchi et al., 1991 [12]	Japan	2 m to 11 y	1 yr	20	-	16 (80)	5 (25)	15 (75)	1 (5)	-	3 (15)	3 (15)	5 (25)
Matsuzaki et al., 2006 [1]	Japan	<1 to 13 y	-	170	29 (17)	153 (90)	105 (62)	126 (74)	21 (12)	10 (6)	17 (10)	24 (14)	-
Gouarin et al., 2008 * [101]	France	4 m to 74 y	-	18	12 (67)	13 (93)	8 (57)	10 (71)	2 (14)	-	4 (29)	5 (36)	-
Anton et al., 2011 [104]	Spain	1 to 60 y	20.5	12	-	10 (83)	-	10 (83)	-	4 (33)	-	-	-
Salez et al., 2014 [77]	France; Reunion Island; UK	<1 to 49 y	3.2	12	-	9 (75)	5 (42)	7 (58)	-	-	-	-	4 (33)
Howard et al., 2017 [63]	Peru	<3 y	-	39	-	24 (62)	35 (90)	34 (87)	0	-	-	-	1 (3)
Thielen et al., 2018 [103]	United States	<6 m to >18 y	1.7	70	59 (84)	33 (47)	29 (41)	42 (60)	16 (23)	6 (9)	8 (11)	20 (29)	.

* 14 patients had available clinical information; - = not reported

**Table 2:** Summary of co-infections seen in patients with influenza C virus (ICV) infection.

Pathogen	N
Rhinovirus/Enterovirus	20
Respiratory syncytial virus	16
Adenovirus	11
Influenza A virus	6
Influenza B virus	4
Parainfluenza virus (1–4)	9
Human metapneumovirus	6
Coronavirus (229E, NL63)	3
Rotavirus	2
*Chlamydia pneumoniae*	1
*Moraxella catarrhalis*	1
*Bordetella parapertussis*	1
Mumps virus	1
Rubella virus	1
Herpes simplex virus	1
Total ICV (+)	278

Sources: [1,12,101,103]
